# Role of bFGF in Acquired Resistance upon Anti-VEGF Therapy in Cancer

**DOI:** 10.3390/cancers13061422

**Published:** 2021-03-20

**Authors:** Fatema Tuz Zahra, Md. Sanaullah Sajib, Constantinos M. Mikelis

**Affiliations:** Department of Pharmaceutical Sciences, School of Pharmacy, Texas Tech University Health Sciences Center, Amarillo, TX 79106, USA; fatema.zahra@ttuhsc.edu (F.T.Z.); s.sajib@ttuhsc.edu (M.S.S.)

**Keywords:** bFGF, VEGF, angiogenesis, anti-angiogenic therapy, resistance, cancer

## Abstract

**Simple Summary:**

Anti-angiogenic therapies targeting the vascular endothelial growth factor (VEGF) signaling are established in the arsenal of cancer treatments. Despite the expectations, their benefits are temporary in cancer patients, partly due to the compensatory function of other angiogenic growth factors. This review focuses on the role of basic fibroblast growth factor (bFGF), one of the highly implicated players in the emergence of resistance to anti-angiogenic approaches. Here, we summarize data from various tumor types where bFGF is upregulated after anti-angiogenic treatment, the molecular mechanisms involved, and we highlight the current status and future perspectives of multi-target anti-angiogenic drugs for cancer.

**Abstract:**

Anti-angiogenic approaches targeting the vascular endothelial growth factor (VEGF) signaling pathway have been a significant research focus during the past decades and are well established in clinical practice. Despite the expectations, their benefit is ephemeral in several diseases, including specific cancers. One of the most prominent side effects of the current, VEGF-based, anti-angiogenic treatments remains the development of resistance, mostly due to the upregulation and compensatory mechanisms of other growth factors, with the basic fibroblast growth factor (bFGF) being at the top of the list. Over the past decade, several anti-angiogenic approaches targeting simultaneously different growth factors and their signaling pathways have been developed and some have reached the clinical practice. In the present review, we summarize the knowledge regarding resistance mechanisms upon anti-angiogenic treatment, mainly focusing on bFGF. We discuss its role in acquired resistance upon prolonged anti-angiogenic treatment in different tumor settings, outline the reported resistance mechanisms leading to bFGF upregulation, and summarize the efforts and outcome of combined anti-angiogenic approaches to date.

## 1. Introduction

Angiogenesis is the formation of new blood vessels from preexisting ones [1]. It is the outcome of a coordinated series of events, which takes place mostly during development and in certain occasions during adulthood. Angiogenic activity is controlled by a dynamic balance between growth factors and angiogenesis inhibitors. This balance is disrupted in a series of diseases, where dysregulated angiogenesis is primarily responsible or augments the progression of the disease [2]. Among the diseases where angiogenesis is abnormally increased, thus requiring pharmaceutical intervention, is cancer. Therapeutic endeavors against tumor angiogenesis are a field of intense scientific efforts since Judah Folkman’s visionary observation and pioneering work in the 1970s [3]. The boost in the angiogenesis research field emerged a few years later with the isolation and identification of the two best-known growth factors, vascular endothelial growth factor (VEGF) [4,5,6,7] and basic fibroblast growth factor (bFGF or FGF2) [8], followed by the isolation of a series of heparin-binding growth factors shortly after [3]. To date, VEGF’s isoforms and receptors have been the target for the majority of Food and Drug Administration-(FDA)-approved therapies for tumor angiogenesis blockade [9]. Current anti-angiogenic therapies targeting VEGF signaling pathways are classified as anti-VEGF monoclonal antibodies, VEGF-binding proteins, and VEGF receptor (VEGFR) tyrosine kinase inhibitors (TKIs) [10].

Targeting the tumor microenvironment has been considered an attractive approach for tumor therapy, because contrary to the very heterogeneous cancer cells, stromal cells are considered relatively homogeneous [11]. Preclinical studies with anti-VEGF approaches demonstrated promising results in tumor angiogenesis and permeability inhibition [12,13]. Shortly after, clinical trials with the anti-VEGF monoclonal antibody Bevacizumab as monotherapy or combination therapy were initiated, highlighting the benefit of anti-angiogenesis therapy as cancer treatment for many malignancies [14]. However, in most cases this benefit was assessed in terms of disease-free survival and not overall survival. Thus, with the exception of some indications, such as metastatic colorectal cancer, the final outcome of clinical trials has not met the expectations [15,16,17]. Bevacizumab was FDA-approved in February 2004 as a first line treatment for patients with metastatic carcinoma of the colon and rectum (CRC) in combination with 5-fluorouracil-based chemotherapy, and in 2006, it was approved as a second line treatment for patients with advanced or metastatic CRC after irinotecan with 5-fluorouracil-based chemotherapy [18]. To date, more than ten anti-angiogenic drugs, antibodies or tyrosine kinase inhibitors have been FDA-approved for the treatment of a variety of cancers including glioblastoma, lung, colorectal, renal and breast cancers [19]. However, despite the increasing number of anti-angiogenesis inhibitors and the several years of clinical experience since the approval of bevacizumab, the response to anti-VEGF therapies is still moderate and not outstanding. The reason is the ephemeral effects of anti-angiogenic drugs with limited prolongation of overall survival, which is only seen in some cancers [9].

There are several potential variants for the poor outcome of anti-angiogenic therapies in clinical practice, such as the stage of the primary tumor, the level of vessel maturation, differential VEGF expression, differentiated anti-angiogenic drug efficacy in the presence of chemotherapy and the differential genetic identity of tumor endothelial cells, to name a few [9,16]. Apart from the VEGF family, several other growth factors either mediate distinct functions of the angiogenic process or act synergistically [2]. One of the major reasons for the limited outcome of anti-angiogenic therapies is “evasive resistance”, which refers to the alternative pathways that are activated upon the blockade of a specific angiogenesis pathway [20]. The outcome of evasive resistance, where the specific anti-angiogenic target remains inhibited, is adaptive response, which differs from the traditional drug resistance or intrinsic non-responsiveness, the other resistance mechanism, where the inhibition of the anti-angiogenic target is not achieved due to mutational alteration of the target or alterations in drug uptake and efflux [21]. 

Resistance to the VEGF/VEGFR signaling inhibitors has been attributed to the activation of alternative pro-angiogenic signaling pathways in the tumor or tumor microenvironment. A variety of other cell types, such as bone marrow-derived cells, fibroblasts and monocytes express a plethora of alternative angiogenic factors such as basic fibroblast growth factor (bFGF), angiopoietins, platelet-derived growth factor (PDGF) and epidermal growth factor (EGF), which can substitute for VEGF. Among these alternative growth factors, bFGF has been widely considered a major player in anti-angiogenic tumor resistance mechanisms, with other growth factors to follow [11,16,21]. In this review, we will discuss the role, preclinical, clinical evidence and molecular pathways triggered by bFGF-driven resistance to anti-VEGF therapy.

## 2. Basic Fibroblast Growth Factor (bFGF): A Pro-Angiogenic Growth Factor

The FGF family in mammals consists of 18 secreted glycoproteins [22], which signal through the FGF receptors (FGFRs). The FGFRs comprise four transmembrane receptor tyrosine kinases FGFR1, FGFR2, FGFR3 and FGFR4 which get auto-phosphorylated upon the binding of FGF members on different types of cells [23,24]. The extracellular domain of the FGFRs contains three immunoglobulin-like (Ig-like) domains, which present structural variability and thus ligand binding specificity due to alternative splicing [25,26]. The role of the FGF/FGFR family during development and adulthood is pivotal. During development it regulates mesoderm patterning and organogenesis [27,28] and in adults it regulates angiogenesis-related functions, such as wound healing [22]. Gain- or loss-of-function mutations of the FGFR family are driving forces of several pathological conditions, highlighting them as targets for pharmaceutical intervention [22]. In cancer, the FGF/FGFR family regulates cellular proliferation, differentiation, apoptosis, angiogenesis and inflammation through different mechanisms, including aberrant expression, mutation and gene amplification [29,30,31,32]. The classical FGF signaling can be transduced by RAS/MAPK, PI3K/Akt, Src tyrosine kinase and STAT pathways, which consist targets of current anti-cancer approaches [29,32,33].

Among the FGF family members, bFGF constitutes the prototypic and best characterized pro-angiogenic factor. The expression of bFGF is increased at sites of chronic inflammation [34,35,36], after tissue injury [37], and in different types of human cancers [38]. Among the members of the FGF1 subfamily, FGF1 can bind all FGFRs whereas bFGF has preference to the c isoforms of FGFR1, FGFR2 and FGFR3 [39,40]. Among the FGFRs, FGFR1, FGFR3 and less frequently FGFR2 are found in endothelial cells (ECs) with minimal or no expression of FGFR4 [26,41]. Upon binding with its receptors on ECs, bFGF can directly promote angiogenesis in vitro and in vivo [22,42,43]. In vivo, bFGF is able to induce neovascularization in a variety of animal models, such as the chick embryo chorioallantoic membrane (CAM) assay, the rodent cornea assay, the subcutaneous matrigel plug assay in mice, and the zebrafish yolk membrane assay [38,44]. bFGF can act on endothelial cells via a paracrine mode of action released by tumor stromal and inflammatory cells and/or by mobilization from the extracellular matrix (ECM). On the other hand, bFGF can also be produced endogenously by ECs and induce angiogenesis via autocrine, intracrine or paracrine manners [38,45]. However, bFGF deficiency, double FGF1 and bFGF deficiency, as well as bFGF overexpression did not lead to lethality due to vascular defects, which can be explained by the presence of compensatory mechanisms in the vascular system [22,38].

Several studies have confirmed the integration of angiogenesis and inflammation in a number of physiological and pathological conditions, including cancer [46,47,48,49]. bFGF-mediated angiogenesis can be promoted by inflammation [50]. Inflammatory cells can express bFGF and inflammatory mediators can activate the endothelium to synthesize and release bFGF, which in turn stimulates angiogenesis through an autocrine manner. The inflammatory response can also increase bFGF production and release by causing cell damage, fluid and plasma protein exudation, and hypoxia [51,52]. On the other hand, bFGF can amplify the inflammatory and angiogenic response by interacting with endothelial cells. Gene expression profiling has revealed a pro-inflammatory signature of bFGF-stimulated murine microvascular endothelial cells characterized by the up-regulation of pro-inflammatory cytokines/chemokines and their receptors, endothelial cell adhesion molecules, and members of the eicosanoid pathway [51]. Macrophages are a source of bFGF and express FGFRs. Monocytes/macrophages play a functional, non-redundant role in bFGF-mediated angiogenesis revealed from early recruitment of mononuclear phagocytes preceding blood vessel formation in bFGF-driven angiogenesis in the matrigel plug assay, while in tumors, increased bFGF regulates macrophage polarization [51,53]. Apart from the pro-inflammatory signature, bFGF also contributes to the increased expression of a variety of pro-angiogenic growth factors in the endothelium, including itself, VEGF and angiopoietin-2 (Ang2) [51,54,55,56]. Overall, bFGF contributes to the modulation of the neovascularization process triggered by growth factors via activating an autocrine loop of amplification of the angiogenic response and by paracrine activity exerted by endothelium-derived cytokines/chemokines on inflammatory cells [57].

## 3. bFGF in Cancer: A Prominent Resistance Mechanism upon Anti-Angiogenic Therapy

Targeting tumor-induced angiogenesis has mostly focused on the VEGF signaling pathway, and was implemented more than 15 years ago with the introduction of bevacizumab, a humanized, recombinant monoclonal antibody against VEGF-A [58]. By binding to circulating, soluble VEGF-A, bevacizumab inhibits its interaction with VEGFR2 and the activation of the downstream signaling pathways. Thus, it provides anti-tumor effectiveness by inhibiting angiogenesis and microvascular density, inducing the regression of newly formed vessels. An important and more recent goal of antiangiogenic therapies is vascular normalization. Normalizing the tumor vasculature renders the tumor susceptible for anti-cancer therapy or immunotherapy [59,60]. Despite the encouraging preclinical data for anti-VEGF therapy and the clinical success in other angiogenesis-related pathologies, such as age-related macular degeneration [61], the clinical outcome in cancer treatments did not meet the expectations. Bevacizumab has been approved since 2004 and is currently marketed in 134 countries worldwide for a number of solid tumors [60], thus there is an increasing number of studies denoting the upregulation of bFGF as an important resistance mechanism, contributing to the ephemeral nature of anti-angiogenic results, important examples of which we highlight below and are summarized in Table 1.

### 3.1. Glioblastoma

Bevacizumab in combination with temozolomide has been approved for newly diagnosed and recurrent malignant glioma in the United States and other countries and provides the clinically meaningful prolongation of progression-free survival (PFS) and non-detrimental increase in overall survival (OS) [60,78]. In a case study, this treatment led to dramatic but transient tumor reduction, and tumor analysis upon recurrence demonstrated VEGF signaling blockade but upregulation of matrix metalloproteinases (MMPs) and sustained p44/42 phosphorylation, denoting the activation of compensatory mechanisms [62]. Immunohistochemical staining in four autopsied malignant gliomas showed increased proliferation in CD31(-)/SMA(+) pericytes around tumor vessels after bevacizumab treatment and no significant changes in the number of tumor vessels in initial and autopsied tumor vessels before and after bevacizumab administration. VEGF-A was present in all tumors at the initial surgery, but its expression was reduced after bevacizumab administration. Interestingly, bFGF and PDGF expression was increased in the endothelial cells, pericytes and tumor cells upon bevacizumab treatment, indicating that the inhibition of VEGF alone is not sufficient to maintain the inhibition of neovascularization due to resistance by bFGF and pericyte coverage by PDGF. The molecular mechanism of bFGF upregulation upon bevacizumab treatment, although not delineated, was speculated to be a result of negative feedback due to the continuous inhibition of the VEGF-driven angiogenic pathway [63]. 

In vitro, although bevacizumab was capable of sequestering the majority of the autocrine secretion of the highly VEGF-expressing U87 glioblastoma and NCS23 glioma stem cells, it induced invasion in a concentration dependent manner [64]. Moreover, it led to bFGF mRNA and protein upregulation in vitro and in vivo, which indicates the potential of glioblastoma cells to escape from antiangiogenic treatment. Consistent with this phenotype, further upregulation of invasion-related proteins, such as matrix metalloproteinases (MMP-2, MMP-9, MMP-12), secreted protein acidic and rich in cysteine (SPARC) and tissue inhibitors of metalloproteinases (TIMPs), allowed the cancer cells to invade into surrounding brain areas in the in vivo glioblastoma xenograft model. The upregulation of bFGF in the glioblastoma xenograft model was further responsible for the rapid increase in vascularity and cellular proliferation, denoting resistance development after the long-term antiangiogenic treatment. Mechanistically, bFGF upregulation was hypoxia-driven, since the hypoxia markers hypoxia-inducible factor 2a (HIF-2a) and carbonic anhydrase IX (CA IX) were also increased. In the U87 xenograft model, after short term (4 weeks) VEGF blockade, bFGF levels were not increased and microvessel density was significantly reduced, but as VEGF blockade continued (7 weeks) bFGF levels increased, similar to the in vitro study, along with microvessel density and tumor cell proliferation, indicating the reactivation of angiogenesis [64]. 

### 3.2. Head and Neck Squamous Cell Carcinoma (HNSCC)

bFGF upregulation appears to be an important resistance mechanism upon bevacizumab treatment in head and neck squamous cell carcinoma (HNSCC). Through an HNSCC xenograft model of acquired resistance to bevacizumab, it was demonstrated that bevacizumab-resistant tumors maintained angiogenesis and prevented endothelial apoptosis, despite the sequestration of VEGF. Whole genome microarray analysis revealed the upregulation of angiogenesis-related genes including bFGF, FGFR1-3, PLCg2, FZD4, CX3CL1 and CCL5 in the bevacizumab-resistant tumor cells. The fact that bevacizumab led to the overexpression of several members of the FGF/FGFR family, including bFGF and FGFR1-3, as well as the activation of downstream signaling effectors including PLCg1, PLCg2, AKT and ERK, strengthens the involvement of the FGF axis in bevacizumab-associated resistance in the HNSCC xenograft model. Co-targeting of the VEGF and FGF pathways led to the restoration of sensitivity to anti-VEGF therapy in bevacizumab-resistant tumors, demonstrating that the upregulation of FGF/FGFR autocrine signaling plays a crucial role in circumventing VEGF inhibition in bevacizumab-resistant tumor cells [30]. 

### 3.3. Gastric Cancer

In human gastric cancer xenograft models, bFGF expression was proposed as a biomarker for antitumor activity of bevacizumab. Refractory to bevacizumab treatment models presented high bFGF levels and the VEGF/bFGF ratio provided a more accurate correlation of sensitivity to bevacizumab, than VEGF expression itself [66]. Irrespective from its role in the vascular system, the deregulation of the FGFR pathway, through point mutations, gene fusions or ligand overexpression, has been recently considered an oncogenic driver for gastrointestinal stromal tumors [79]. It was recently reported that the higher response of MKN45 than SNU5 gastric cancer cells to Pazopanib, a tyrosine kinase inhibitor that targets VEGFR1-3, PDGFRα,β, c-KIT, FGFR1-4 and CSF1R, was due to the higher FGFR2 and FGFR3 expression. The sensitivity of MKN45 cells was higher in the in vivo compared to the in vitro settings, which was attributed to the lower expression of FGF-binding protein 1 (FGFBP1) in the in vitro setting. FGFBP1 mediates the release of bFGF from the extracellular matrix, thus highlighting the FGF signaling as an important mediator for pazopanib treatment. Although the MKN45 xenografts were initially responsive to pazopanib, they later transitioned to a mesenchymal-like phenotype, becoming more invasive and developing resistance, which led to tumor regrowth after drug withdrawal [65]. 

### 3.4. Colorectal Carcinoma

The stimulating role of bFGF on colorectal carcinoma cell invasion is long established [80]. Cytokine analysis in metastatic colorectal cancer patients undergoing a phase II [67] clinical trial of bevacizumab and FLORFIRI+B treatment regimen revealed an increment of bFGF levels in the plasma of a subset of patient population during the emergence of resistance. The FLORFIRI+B regimen contained bevacizumab, irinotecan, bolus fluorouracil and leucovorin, followed by infusion of fluorouracil. Although the mean bFGF levels decreased after one cycle of FLORFIRI+B, they increased before and at the time of disease progression [67], indicating the participation of bFGF in resistance mechanisms. VEGF downregulation in endothelial cells isolated from tumors of colon cancer patients led to significant bFGF upregulation, further highlighting the impact of the tumor vascular endothelium in bFGF-dependent compensatory mechanisms [68].

### 3.5. Pancreatic Cancer

In a murine model of islet cell carcinogenesis, qRT-PCR analysis from total tumor mRNA revealed the upregulation of several FGF members, including bFGF, upon VEGFR2-blocking treatment, which was further confirmed by ELISA. Although bFGF was upregulated both in tumor cells and tumor endothelial cells, expression of FGFR1 and FGFR2 was not affected in this model. The trigger for bFGF upregulation was the increased levels of tumor hypoxia after VEGFR2 inhibition, which was also confirmed in the RIP-Tag2 tumor-derived βTC3 cell line under hypoxia in vitro. In the same model, and contrary to the in vivo data, the FGF1 levels remained unaffected [69]. 

### 3.6. Liver Cancer

Hepatocellular carcinoma is the most common type of liver cancer and occurs frequently in patients with liver cirrhosis or chronic liver diseases. Anti-VEGF treatment increases survival and is the standard-of-care for hepatocellular carcinoma (HCC), with sorafenib (VEGFR2, PDGFR, Raf1 inhibitor), lenvatinib (VEGFR, FGFR, c-Kit and RET inhibitor) and regorafenib (VEGFR2, Tie2 inhibitor) being common treatments [81,82]. The plasma levels of VEGF and bFGF in hepatocellular carcinoma patients are increased with the progression of the disease, upregulating PD-1 expression and inducing immune suppression [70].

Tumor vessel normalization, a major goal of anti-angiogenic treatments, was achieved in liver cancer with the combined inhibition of VEGFR and FGFR pathways. An elegant study demonstrated that combined VEGFR and FGFR inhibition potentiated the efficacy of anti-PD-1 treatment, inducing vessel normalization and antitumor efficacy [70].

### 3.7. Renal Cell Carcinoma

Renal cell carcinoma (RCC) is a highly vascularized tumor, thus tumor angiogenesis plays a critical role in the development of metastatic RCC. Several anti-angiogenic drugs have been approved for RCC treatment in the United States, including bevacizumab, sunitinib, pazopanib and sorafenib [19,83,84]. While angiogenesis targeting via VEGF blockade is the standard of care in metastatic RCC, around 20% of the patients do not respond to the treatment. For the rest, although they gain initial benefits from anti-angiogenic therapy, they eventually develop resistance between 6 and 15 months of treatment, which is attributed to revascularization, driven by the tumor microenvironment [85]. Sunitinib treatment of RCC patients led to an increase in serum bFGF levels, irrespective of the treatment outcome, although patients with no response to sunitinib presented higher bFGF levels than the ones with a temporary clinical benefit or a better response [71]. These data are consistent with previous clinical findings demonstrating that bFGF is responsible for sunitinib resistance, indicating the necessity of targeting both VEGF and bFGF pathways simultaneously [72,73]. Patients under anti-VEGF therapy can still present beneficial outcome by a multi-kinase inhibitor, such as sorafenib. When sunitinib-resistant patients were treated with sorafenib the overall survival was improved, revealing both the importance of the proper timing and order of each targeted approach [72,86].

### 3.8. Breast Cancer

In breast cancer cells, the role of VEGF is indispensable for the initial tumor growth, but bFGF upregulation can compensate for the VEGF downregulation at later stages. This was elegantly demonstrated by Tet-regulated VEGF expression in the T-47D breast cancer cells. VEGF downregulation was detrimental for tumor inoculation or early tumor growth, however, upon VEGF suppression at later stages, bFGF expression was upregulated without affecting tumor growth. bFGF was not detectable in tumors of the same size overexpressing VEGF during the entire experimental period [75].

Tumor growth directly depends on the tumor microenvironment, and obesity, as a systemic condition associated with hypoxic adipose tissues, affects the tumor microenvironment, regulating tumor growth and outcome of anti-cancer therapeutic approaches. It was recently shown that the plasma concentration of bFGF is higher in obese breast cancer patients. Adipose tissue size inversely correlated with vascular density and bFGF overexpression was particularly abundant in adipose-rich tissues based on the immunohistochemical observation of human breast tumor samples from obese patients. Additionally, obesity has been inversely correlated with the response to anti-VEGF treatment. Similarly, the baseline bFGF levels were higher in untreated obese compared to untreated lean mice and anti-VEGF treatment increased them further. bFGF overexpression was identified in the adipocyte-rich tumor periphery and in activated cancer-associated fibroblasts, which is consistent with bFGF localization in adipocyte-rich human breast cancer. In two syngeneic breast cancer tumor models, it was demonstrated that tumors were less vascularized and more hypoxic and anti-VEGF therapy was less potent in reducing vessel density in obese, compared to lean mice. FGF receptor blockade with AZD4547, a pan-FGFR inhibitor, improved tumor responsiveness to anti-VEGF treatment in obese mice, not in lean mice, but showed toxicity [74]. Instead, metformin, a safe and popular anti-diabetic drug, previously shown to reduce cellular bFGF expression and with anti-cancer effect in obese settings [87,88], reduced vessel density and re-sensitized to anti-VEGF therapy in obese mice. Mechanistically, metformin treatment reduced bFGF mRNA and protein expression and inhibited bFGF downstream signaling pathways, such as AKT, S6, ERK and STAT3 [74].

### 3.9. Cervical Carcinoma

The role of pericytes is equally important to the one of endothelial cells in angiogenesis, as they provide survival signaling to endothelial cells and play an important functional role in mediating blood flow and endothelial cell permeability [89]. Similarly, in tumors, the inhibition of VEGF signaling leads to the reduction in immature (without pericyte coverage) tumor microvasculature with an increase in the percentage of vessels with pericyte coverage (mature vessels) [89,90]. The PDGF/PDGFR signaling is the predominant mediator of pericyte migration and proliferation [91]. bFGF shared the same expression pattern with the PDGF receptor in stromal fibroblasts in a genetically engineered model of cervical carcinogenesis and their expression was increased in cancer-associated fibroblasts (CAFs), but not in other cell types. Moreover, bFGF was demonstrated to be a downstream effector in PDGF signaling, as its expression was decreased upon treatment with the selective PDGFR inhibitor imatinib in cervical carcinoma [76]. Therefore, bFGF plays a key regulating role in PDGF-induced angiogenesis and in acquired resistance induced by VEGF-targeted therapy [76,89].

### 3.10. Prostate Cancer

Prostate cancer is considered one of the resistant cancers to anti-angiogenic treatments and one of the reported reasons is the involvement of the FGF-FGFR family in transformation and angiogenesis [10]. VEGF overexpression and microvessel density have been associated with tumor growth, poor prognosis and increased metastatic potential. In phase II clinical trials of castration-resistant advanced prostate cancer, anti-angiogenic therapy improved relapsed-free survival and led to disease stabilization, whereas in phase III trials, no significant outcome was identified in terms of overall survival. Instead, anti-angiogenic treatment caused increased toxicity and greater incidence of treatment-related death [92]. The trials included anti-VEGF antibodies, such as bevacizumab, decoy receptors, such as aflibercept, as well as tyrosine kinase inhibitors, such as sunitinib (targeting VEGFR2, PDGFRβ, c-Kit and RET) [77,92,93]. bFGF, along with interleukin-6 (IL-6) are known contributors of androgen ablation, chemotherapy resistance and metastatic dissemination of prostate cancer cells. It was further shown that bFGF triggers IL-6 and prostate-specific membrane antigen (PSMA) expression, markers of chronic inflammation and prostate cancer prostate cancer progression in advanced stages [94]. Finally, the high FGFR expression levels of the prostate cancer cells have been taken into consideration for the design of tumor cell- and tumor endothelial cell-specific liposomes for improved doxorubicin delivery, with promising results [95,96].

## 4. Mechanisms of bFGF Release or Upregulation with Angiogenic Potential

**Figure 1:**
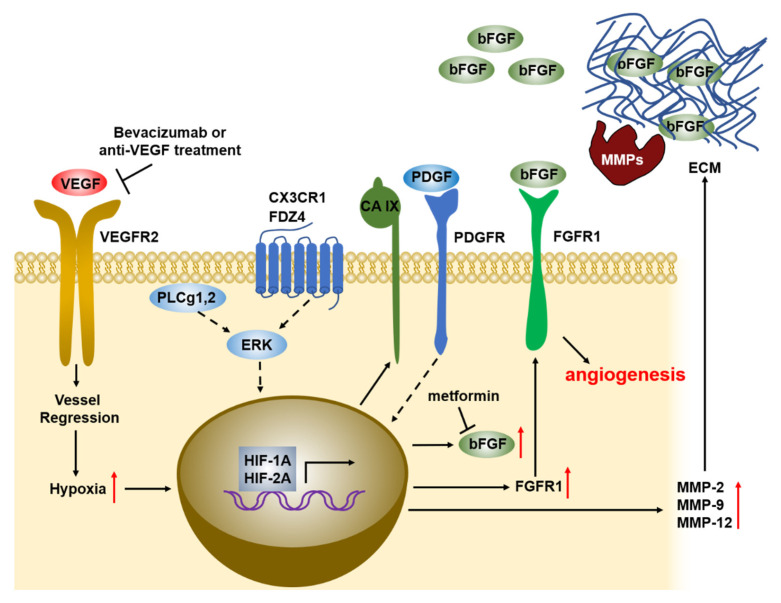
Pathways of bFGF-induced compensation upon anti-VEGF treatment. Bevacizumab or anti-VEGF treatment leads to vascular regression, inducing hypoxia in the surrounding tissues. Hypoxia drives the expression of carbonic anhydrase IX and activates HIF-1A and HIF-2A, increasing bFGF levels. Metformin treatment blocks bFGF mRNA and protein levels. A similar increase in bFGF levels is achieved upon anti-VEGF treatment in cancer cells, via the upregulation of PLCg1,2, FDZ4 and CX3CL1 (ligand of CX3CR1) with a subsequent ERK activation. PDGFR activation in smooth muscle cells leads to FGFR1 expression. HIF-2A activation induces the expression of MMP-2, -9 and -12, releasing bFGF molecules via extracellular matrix (ECM) degradation.

The origin and release mechanisms of the bFGF pool driving angiogenesis has been well-reported (Figure 1). Most of the endothelial-synthesized bFGF remains cell-associated, however, a portion of the bFGF pool is sequestered in the subendothelial extracellular matrix (ECM) for deposit [97]. Like the other FGFs, bFGF is a heparin-binding molecule, bound to heparan sulfate, which constitutes more than 90% of the subendothelial ECM glycosaminoglycan side chains and serves as a sink to concentrate and stabilize bFGF, protecting it from degradative enzymes. The endothelial cells also synthesize secreted or cell membrane- and extracellular matrix (ECM)-associated heparan sulfate proteoglycans (HSPGs) [98,99]. HSPGs and heparan sulfate protect bFGF from thermal denaturation and proteolytic degradation and further modulate bFGF activity. Moreover, bFGF binding to HSPGs serves as a reservoir, from which FGFs can be released in response to specific triggering events [98,100,101]. The interaction of bFGF with HSPG modulates FGF activity by increasing its receptor binding affinity with the establishment of stable growth factor-receptor complexes and the facilitation of FGFR dimerization with subsequent activation [102,103]. Release from the ECM storage takes place after injury, mild perturbation of endothelial cells or release of proteases, further stimulating the autocrine proliferation of adjacent endothelial cells and leading to angiogenesis [97,104]. One of the drivers of diseases characterized by aberrant angiogenesis, such as choroidal neovascularization, is the increased ECM cleavage and subsequent release of bFGF [105]. During tumor-induced angiogenesis, the release of bFGF is partly regulated by the activity of tumor-derived heparan sulfate-degrading enzymes, which release bFGF in the capillary basement membrane [104,106]. 

One group of these enzymes are the MMPs, a family of soluble and membrane-anchored proteolytic enzymes which can degrade components of ECM. It is well-established that MMPs are important regulators of angiogenesis, as they break down matrix components and thus clear the path for migrating ECs during angiogenesis. Additionally, MMPs can also switch on angiogenesis by liberating matrix-bound bFGF [105,107,108]. MMP-2 expression has been correlated with the de novo formation of small capillaries in tumors. Bevacizumab treatment led to increased expression and enzymatic activity of MMP-2 and MMP-9, common metalloproteinases associated with neovascularization of tumors, in glioblastoma cells both in vitro and in vivo [64,109]. Bevacizumab treatment also resulted in the upregulation of bFGF and of the MMP inhibitors TIMP-1 and TIMP-2, as a potential response to MMP upregulation in U87 and NSC23 glioblastoma cells, suggesting that tumors can overcome anti-VEGF treatment via the release of bFGFs from ECM with the help of MMPs, supporting an autocrine pattern of bFGF signal transduction that results in neovascularization [64].

There is an intimate, but well-characterized crosstalk between bFGF and the different members of the VEGF family during angiogenesis, lymphangiogenesis and vasculogenesis. Among the members of the VEGF family, VEGF-A/VEGFR2 appears to play a major role in blood vessel angiogenesis and VEGF-C and VEGF-D are involved in lymphangiogenesis by interacting with VEGFR3 [38,110,111]. Previous studies have the reported synergistic and complementary activity of bFGF with VEGF and PDGF-BB [112,113,114]. bFGF upregulates PDGFR expression to increase the responsiveness to PDGF-BB in endothelial cells and PDGF-BB-treated vascular smooth muscle cells may contribute to the increased responsiveness to bFGF by upregulating FGFR1 expression [114,115]. In turn, bFGF can also contribute to the increased expression of other proangiogenic factors, highlighting the complex compensatory mechanisms that regulate angiogenic processes and contributing to resistance upon anti-VEGF treatment [11,69].

The most common and widely accepted mechanism of bFGF upregulation upon VEGF inhibition is related to the induction of tumor hypoxia. Antiangiogenic therapy in different tumor types induces the elevation of hypoxia markers HIF-1A, HIF-2A and CA IX, followed by increased bFGF expression [10,64]. In bevacizumab-resistant HNSCCs, bFGF upregulation was mediated by ERK, which was induced due to higher expression of upstream activator genes including phospholipase C (PLCg2), frizzled receptor-4 (FDZ4), chemokine C-X3-C motif (CX3CL1), and chemokine C-C motif ligand 5 (CCL5). This was confirmed by the decreased activation of ERK and the corresponding decrease in bFGF levels upon the downregulation of each of these genes [30]. 

## 5. Targeting Anti-VEGF Resistance: Combinatorial Therapies

**Figure 2:**
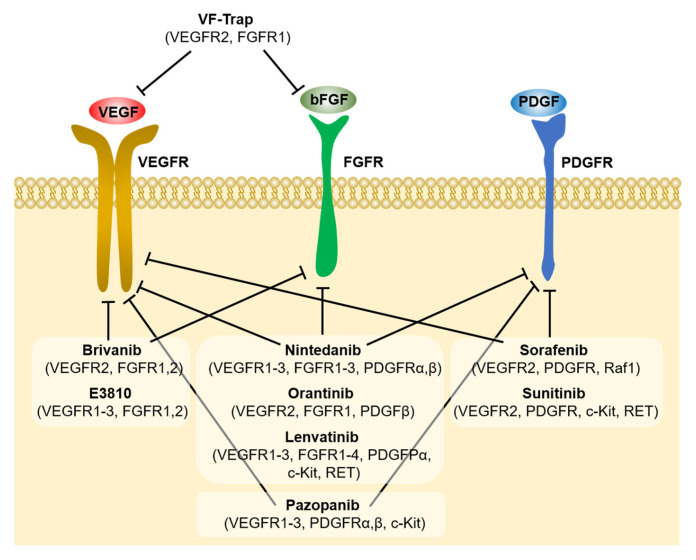
Inhibitors targeting combination of angiogenesis pathways either blocking ligand interaction (VF-Trap) or downstream signaling pathways. The parenthesis below each inhibitor highlight the growth factor receptors targeted by each inhibitor.

As bFGF is a prominent factor in anti-VEGF therapy resistance, experimental evidence suggests that targeting bFGF in addition to VEGF may provide synergistic outcome and prove beneficial for the treatment of angiogenesis-related diseases, including cancer. Different chemical structures and mechanisms of action of several bFGF inhibitors have been described (Figure 2). One soluble pattern recognition receptor long-pentraxin-3 (PTX3), which binds with bFGF with high affinity and specificity, has been shown to antagonize bFGF activity. This interaction leads to the inhibition of the angiogenic activity of bFGF, as it can no longer bind with FGFRs, ultimately blocking bFGF-mediated tumor angiogenesis and growth. PTX3 has a unique N-terminal extension which has been identified as a bFGF binding domain. PTX3-derived synthetic peptides have shown significant anti-angiogenic activity in vitro and in vivo, with potential implications in cancer therapy [57]. 

Growing evidence suggests that the dual inhibition of VEGFR and FGFR in preclinical models can overcome anti-VEGF therapy resistance. Pancreatic islet carcinogenesis was one of the first models where the FGF family of ligands was identified to be among the primary resistance mechanisms [69,89]. Treatment with an anti-VEGFR2-blocking monoclonal antibody decreased the vascular density after 10 days in the RIP-Tag2 mouse model of islet cell carcinogenesis. However, an angiogenic rebound in tumors at 4 weeks of treatment was noted, which was associated with an increase in bFGF expression. The concomitant blockade of VEGF signaling with the VEGFR2-blocking monoclonal antibody and FGF signaling by adenovirus-delivered soluble form of FGFR2 (FGF-trap) significantly reduced tumor burden and vessel density compared to the anti-VEGFR2 alone [69]. Co-targeting VEGF by bevacizumab and FGFRs by the small molecule inhibitor PD173074 abrogated tumor growth in the bevacizumab-resistant HNSCC xenograft model by inhibiting tumor angiogenesis [30].

A novel chimeric decoy receptor VF-Trap fusion protein that binds both VEGF and bFGF was developed by Li et al. to simultaneously block activity of both VEGF and bFGF pathways and achieve an additive anti-tumor effect. In vitro, VF-trap blocked VEGF- and bFGF-induced vascular endothelial cell proliferation and migration, while in vivo, combined VEGF and bFGF sequestration resulted in a significant inhibition of renal and lung xenograft tumor growth compared to the single VEGF inhibition [116]. 

The efforts for the combined blockade of VEGF and FGF pathways have led to the development of tyrosine kinase inhibitors, which unlike the antibodies, target the downstream signaling pathways of VEGF, FGF and other growth factors, with brivanib and E-3810 being characteristic examples. Brivanib is a tyrosine kinase inhibitor that targets VEGFR2, FGFR1 and FGFR2 [117,118]. In preclinical studies, brivanib administration demonstrated encouraging results in different cancer models, but it mostly led to tumor inhibition rather than tumor regression and its efficacy depended on endogenous bFGF expression [117,118]. In the clinical setting, brivanib in combination with standard chemotherapy and monoclonal antibodies demonstrated moderate and manageable side effects and provided encouraging results for hepatocellular carcinoma and colorectal cancer, increasing progression-free survival [118]. Unfortunately, in a phase III clinical trial for unresectable hepatocellular carcinoma, brivanib in combination with chemotherapy failed to improve overall survival. In fact, this multinational study was terminated earlier, when two other phase III studies with brivanib on advanced HCC patients failed to meet their overall survival objectives [119]. Interestingly, in a recent case report, brivanib demonstrated excellent antitumor efficacy for an HCC patient as second-line therapy, bringing up the possibility of a better clinical outcome of brivanib after HCC resection, with long-term treatment and the delayed onset of administration. Specifically, brivanib was administrated as a monotherapy to a patient who had developed lung metastases one year after HCC resection, and after sorafenib treatment for three months failed to hinder disease progression. A period of 2.5 months after brivanib treatment, lung metastases decreased or disappeared and lymph node metastases decreased, a trend that continued at later evaluations. The total duration of brivanib treatment was 11 months due to grade 2 thrombocytopenia, but with tolerable side effects, and 4 months after the end of treatment the patient remained in good condition without signs of deterioration. This could suggest that brivanib may be more effective with long-term treatment in a delayed-onset fashion [120]. In a meta-analysis, the efficacy of brivanib in combination with cetuximab and chemotherapy was found to be better than the efficacy of the combination of cetuximab with chemotherapy or sorafenib with chemotherapy, although it presented toxicity. The superiority of this combination could be explained due the simultaneous inhibition of VEGF-induced angiogenesis in the endothelial cells with the EGFR signaling blockade in the tumor cells [121]. E3810 is a tyrosine kinase inhibitor that targets VEGFR1, VEGFR2, VEGFR3, FGFR1 and FGFR2 and retrieves responses in tumors that are not responsive to other small inhibitors, such as sunitinib. In preclinical studies, E3810 showed tumor regression and significantly delayed tumor growth, although tumors resumed their growth when treatment was suspended [122].

Sorafenib and sunitinib are other prominent members of this group, that have been tested, shown to increase progression free survival in a variety of cancers and are FDA-approved. Sorafenib targets VEGFR2, PDGFRβ and Raf1 kinase activity and sunitinib targets VEGFR2, PDGFRβ, c-Kit and RET [20]. Even these, however, have not increased the overall survival significantly [16,20]. Similarly, nintedanib, a tyrosine kinase inhibitor that blocks the VEGF, FGF and PDGF pathways has been approved for non-small-cell lung cancer and recently for idiopathic pulmonary fibrosis [123,124,125], while pazopanib, a small molecule multi-kinase inhibitor that blocks VEGF, FGF, PDGF pathways and c-Kit and has been approved for advanced soft-tissue sarcoma and renal cell carcinoma [126]. Orantinib (SU6668) is another small molecule inhibitor that binds and inhibits the phosphorylation of VEGFR2, FGFR1 and PDGFR-β, thus blocking the signal transduction of the corresponding ligands. In vivo, it inhibited the growth of glioma, melanoma, lung, colon, ovarian and epidermoid tumor xenografts and suppressed tumor angiogenesis, by inhibiting tumor endothelial cell survival directly (apoptosis of endothelial and tumor cells) or via inhibition of pericyte coverage [127,128]. In a phase III clinical trial of hepatocellular carcinoma, orantinib increased the time to progression but did not improve the overall survival [129]. Lenvatinib, a VEGFR1-3, FGFR1-4, PDGFRα, c-Kit and RET inhibitor, is one of the six approved systemic therapies for hepatocellular carcinoma, the most common form of liver cancer [130,131]. 

In terms of tumor vessel normalization, the information existing regarding the efficacy of these inhibitors is still limited. It was demonstrated that the effect of lenvatinib with anti-PD-1 treatment was superior to the outcome of single sorafenib or FGFR treatment and improved anti-cancer activity. This was due to inhibition of immunosuppressive effects and the induction of vessel normalization, opening up the potential of combined anti-angiogenic treatments and tumor vascular normalization for immunotherapy [70].

## 6. Conclusions and Perspectives

The last few decades, intense scientific efforts on anti-angiogenic therapies have provided beneficial outcome in the clinical setting for some diseases, while they are still far from the desired therapeutic outcome in others, including cancer. Contrary to the classical notion of vascular regression, the main goal of current anti-angiogenic treatments is tumor vascular normalization and maturity, which provides increased tumor access to chemotherapeutic drugs and higher efficacy of cancer immunotherapy. The variety of cytokines and growth factors, the complexity of their signaling pathways and the interplay and compensation among them have hindered the generation of potent therapies. Targeting several growth factors with combinatory therapies, downstream signaling adaptors, where different growth factor pathways converge, important endothelial functions, such as metabolism, and the induction of vascular normalization remain promising areas that drive the common efforts towards novel anti-angiogenic therapies and cancer treatment. The compensatory mechanisms triggered upon anti-angiogenic monotherapies have driven the establishment of the current multitargeting anti-angiogenic inhibitors in the clinical practice. The identification and potent inhibition of downstream kinases and key signaling molecules where many angiogenic pathways converge could overcome current issues driven by the diversity of angiogenic ligands and receptors and should be the focus of future research. Moreover, the combination of current or future broad-spectrum anti-angiogenic inhibitors with immunotherapy in different cancers bears high potential to significantly advance the outcome of anticancer treatments and provides a promising field for clinical research. 

## Figures and Tables

**Table 1:** Summary of clinical, preclinical and in vitro tumor studies demonstrating that anti-angiogenic inhibition induced basic fibroblast growth factor (bFGF) expression. The cancer type, anti-angiogenic treatment, effect in bFGF expression and observed outcomes of each study are presented. CD31: cluster of differentiation 31; SMA: smooth muscle actin; FGFR: FGF receptors; MMPs: matrix metalloproteinases; SPARC: secreted protein acidic and rich in cysteine; TIMPs: tissue inhibitors of metalloproteinases; PDGF: platelet-derived growth factor; VEGF: vascular endothelial growth factor; RIP-Tag2: rat insulin promoter-1 driven viral SV40 large T-antigen; HUVEC: human umbilical vein endothelial cells; PDGFR: platelet-derived growth factor receptor.

Cancer Type	Model Used	Treatment	Effect on bFGF	Observed Outcomes	References
Glioblastoma	Clinical	Bevacizumab	↑ bFGF in pericytes, endothelial and tumor cells	↓ Vessel density/no difference ↑ CD31(-)/SMA(+) pericytes ↑ MMPs ↑ VEGFR1 ↓ Akt	[62,63]
	Preclinical (U87)	Bevacizumab	↑ bFGF after 7 weeks	↑ Vascularity, cell proliferation ↑ HIF-2a, CA IX	[64]
	In vitro	Bevacizumab	↑ bFGF in U87 and NCS23 tumor cells	↑ Cell invasion ↑ MMP-2, MMP-9, MMP-12 ↑ Collagen IV, CXCL9 ↑ SPARC, TIMPS ↓ Laminin, integrin β_2_, MMP-1	[64]
Head and neck squamous cell carcinoma	Preclinical (Tu138)	Bevacizumab	↑ bFGF, FGFR1-3	- Sustained angiogenesis ↑ PLCg2, FZD4, CX3CL1 ↑ ERK ↓ Endothelial apoptosis	[30]
Gastric cancer	Clinical/ Preclinical (MKN45)/ In vitro	Pazopanib	↓ FGFRP1 (in vitro)	↑ TWIST ↑ CYP2C19, TFF3, PLA2G2A ↓ EGLN2, MIR590, ↓ LCN2, TET1 ↑ Mesenchymal phenotype	[65]
	Preclinical (GXF97, MKN-45, MKN-28, 4-1^ST^, SC-08-JCK, SC-09-JCK, SCH, SC-10-JCK, NCI-N87)	Bevacizumab	↑ bFGF in bevacizumab-resistant tumor cells	↑ Vessel density ↑ Tumor volume	[66]
Colorectal carcinoma	Clinical	Bevacizumab, fluorouracil, leucovorin, irinotecan (FLORFIRI+B)	↑ Plasma bFGF levels	↑ Resistance	[67]
	In vitro	VEGF RNAiBevacizumab	↑ bFGF in endothelial cells from colon tumors	↑ ANG1	[68]
Pancreatic cancer	Preclinical (RIP-Tag2 model)	VEGFR2-blocking antibodies	↑ bFGF in endothelial and tumor cells	↓ Vessel density ↑ Tumor hypoxia, HIF-1α ↑ FGF1, ANG1 ↑ EphA1, EphA2	[69]
Liver cancer	Preclinical (H22)/ In vitro (HUVEC, HEPG2)	Sorafenib	Potential bFGF increase (higher lenvatinib efficacy)	↑ PD1, CTLA-4, Tim-3 ↑ PD-L1 expression	[70]
Renal cell carcinoma	Clinical	Sunitinib	↑ Plasma bFGF levels	↑ HGF, IL-6, IL-8 ↑ PDGF1, ANG1	[71,72]
	In vitro (HUVEC)	Sunitinib	↑ bFGF efficacy, FGFR activation	↑ Angiogenesis	[73]
Breast cancer	Preclinical (E0771, MCaIV)	Anti-VEGF antibody	↑ bFGF in adipocyte-rich tumor periphery ↑ bFGF in cancer-associated fibroblasts	↑ IL-6, IL-12, CXCL1, TNFα ↓ Tumor vasculature ↑ Hypoxia	[74]
	Preclinical (T-47D)	Tet-regulated VEGF expression	↑ bFGF	↑ Tumor growth	[75]
Cervical carcinoma	Preclinical	Imatinib	↓ bFGF in cancer-associated fibroblasts	↓ PDGFR ↓ Angiogenesis ↓ Epithelial proliferation	[76]
Prostate cancer	Clinical	VEGF inhibitors	↑ FGF-FGFR in tumors	↑ Angiogenic pathways	[10,77]
